# Nuclei in motion: movement and positioning of plant nuclei in development, signaling, symbiosis, and disease

**DOI:** 10.3389/fpls.2014.00129

**Published:** 2014-04-03

**Authors:** Anna H. N. Griffis, Norman R. Groves, Xiao Zhou, Iris Meier

**Affiliations:** ^1^Department of Molecular Genetics, The Ohio State UniversityColumbus, OH, USA; ^2^Center for RNA Biology, The Ohio State UniversityColumbus, OH, USA

**Keywords:** arbuscular mycorrhiza, cytoskeleton, KASH, nodulation, pollen tube, root hair, SUN, trichome

## Abstract

While textbook figures imply nuclei as resting spheres at the center of idealized cells, this picture fits few real situations. Plant nuclei come in many shapes and sizes, and can be actively transported within the cell. In several contexts, this nuclear movement is tightly coupled to a developmental program, the response to an abiotic signal, or a cellular reprogramming during either mutualistic or parasitic plant–microbe interactions. While many such phenomena have been observed and carefully described, the underlying molecular mechanism and the functional significance of the nuclear movement are typically unknown. Here, we survey recent as well as older literature to provide a concise starting point for applying contemporary molecular, genetic and biochemical approaches to this fascinating, yet poorly understood phenomenon.

In animals, nuclei are moved and positioned in a myriad of different cell types, developmental processes, and physiological situations. Classic examples of nuclear movement in non-mammalian systems include *Caenorhabditis elegans* hypodermal cell development and *Drosophila melanogaster* eye disk development (reviewed in Starr and Fridolfsson, 2010). Nuclear movement has also been observed during cell division, neuronal migration, epithelium development, and fertilization (reviewed in Gundersen and Worman, 2013). These nuclear movement and positioning events are regulated by the cytoskeleton, often in concert with nuclear envelope bridging LINC complexes, which link the cytoskeletal forces to the nucleoskeleton. Nuclear movement is also integral to several plant processes, including pollen tube and root hair tip growth, trichome development, symbiotic and pathogenic plant–microbe interactions, and response to mechanical and light stimuli (discussed here), as well as symmetric and asymmetric cell division (reviewed in Smith, 2001). However, unlike in animals, little is known about the mechanism of plant nuclear movement. In this review, we survey nuclear movement events in differentiated plant cells, focusing on physiology and development. By aggregating information on nuclear movement from disparate disciplines, especially those where description of nuclear movement has been largely phenomenological, we hope to encourage research into the mechanism(s) of nuclear movement in plants.

## NUCLEAR MOVEMENT DURING DEVELOPMENT

### POLLEN TUBE GROWTH

The longest journey of plant nuclei is the migration of the vegetative nucleus (VN) and sperm cells (SCs) through pollen tubes. There are two types of angiosperm pollen–tricellular and bicellular. Tricellular pollen contains a VN and two SCs, while bicellular pollen contains a VN and a generative cell (GC), which later undergoes mitosis to generate two SCs (McCue et al., 2011). Since angiosperm VNs and GC/SCs are closely associated and often physically connected (McCue et al., 2011), they are termed the male germ unit (MGU; Dumas et al., 1985). The traveling order of the VN and GC/SCs seems to be species-specific. In many angiosperms, the VN precedes the GC/SCs (VN is closer to the growing pollen tube tip; Heslop-Harrison and Heslop-Harrison, 1989a; McCue et al., 2011). In *Amaryllis vittata* pollen, the VN or GC can enter the growing pollen tube first, but the VN always precedes the GC before the GC undergoes mitosis. Similarly, in tobacco (*Nicotiana tabacum*), the VN initially precedes the GC, and later becomes proximal to the dividing GC (Palevitz, 1993).

The molecular mechanism underlying MGU movement has been widely studied, but remains enigmatic. Depolymerizing tobacco pollen microtubules (MTs) impairs MGU movement and causes abnormal F-actin organization, but does not affect pollen tube growth (Kaul et al., 1987; Åström et al., 1995). During *Galanthus nivalis* pollen tube growth, the VN typically precedes the SC (Heslop-Harrison et al., 1988). Similar to tobacco pollen, MT depolymerizing drugs affected this order and increased the distance between the leading nucleus and the pollen tube apex, while higher concentrations additionally increased VN-GC distance (Heslop-Harrison et al., 1988). However, unlike in tobacco, both concentrations affected *G. nivalis* pollen tube growth (Heslop-Harrison et al., 1988). Despite interspecies differences, these studies suggest a MT network is responsible for MGU movement. Early work identified dynein-related polypeptides in tobacco pollen tubes (Moscatelli et al., 1995). However, as the *Arabidopsis* genome contains no dynein heavy chain genes, whether dyneins participate in MT-dependent MGU movement remains an open question (Lawrence et al., 2001).

On the other hand, discrete foci at the VN periphery of tobacco pollen labeled by anti-myosin suggest a role for myosin and F-actin in VN movement (Tirlapur et al., 1995). Supporting this hypothesis, antibodies raised against non-plant myosins identified putative myosins at the periphery of the VN in *Lilium longiflorum* and *Helleborus foetidus* (Heslop-Harrison and Heslop-Harrison, 1989b; Miller et al., 1995). Depolymerizing *L. henryi* F-actin with cytochalasin D caused rapid contraction of the elongated VN, increased separation between the VN and GC, and reduced VN-pollen tube tip distance (Heslop-Harrison and Heslop-Harrison, 1989a). In addition, the *Lotus japonicus* symbiotic mutant *crinkle* has aberrant F-actin organization in pollen tubes and GC movement defects (Tansengco et al., 2004). In spite of these studies, no specific proteins have been implicated in MGU movement, and whether MGU movement is F-actin-dependent and/or MT-dependent remains unresolved.

### TRICHOME DEVELOPMENT

**FIGURE 1:**
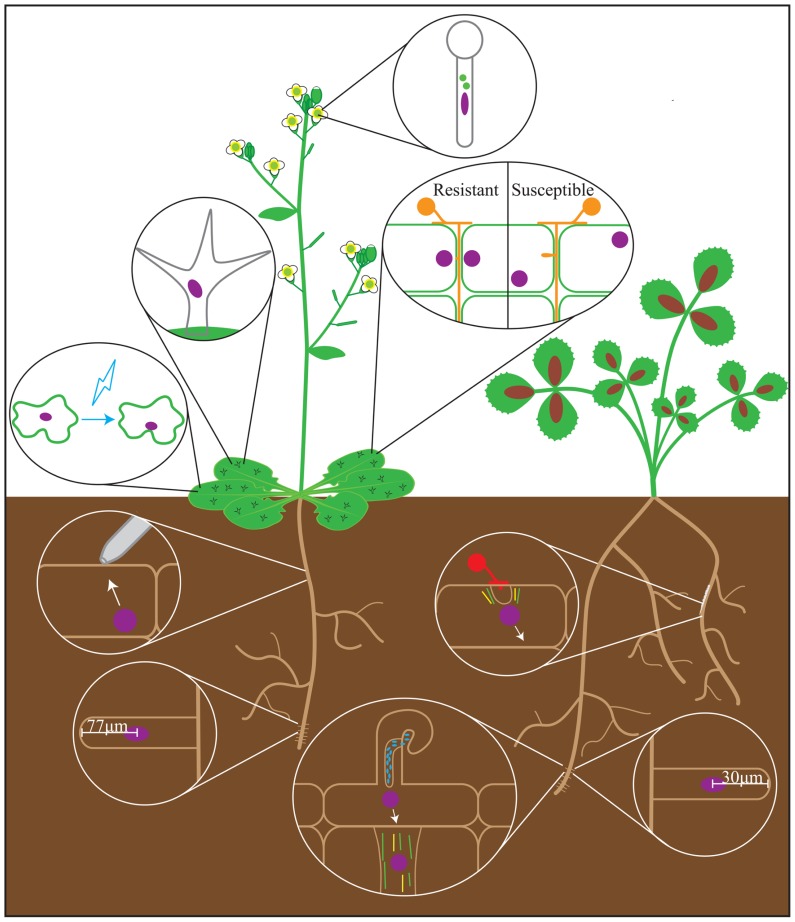
**Nuclear positioning events observed during the plant life cycle**. Depicted are the processes that have been studied in *Arabidopsis* (left) and legumes (represented by *Medicago truncatula,* right) in which nuclear movement may play a role. Processes described in non-legume model plants are shown on *Arabidopsis*. Nuclear positioning events represented are (clockwise from top): pollen tube development, plant-oomycete interactions, arbuscular mycorrhizal symbiosis, root hair development in *Medicago*, nodulation, root hair development in *Arabidopsis*, mechanical stimulation, blue light stimulation, trichome development. Green bars = microtubules; yellow bars = actin cables; red spores = fungi; orange spores = oomycetes; blue ovals = rhizobia; purple ovals = nuclei.

The nucleus is positioned near the primary branch point in mature trichomes, showing that nuclei move during trichome biogenesis (**Figure 1**; Folkers et al., 1997). This is altered in mutants with primary and secondary branch formation defects, and when trichomes are treated with either MT destabilizing or stabilizing drugs (Folkers et al., 1997; Mathur et al., 1999). This correlates with impaired branching of trichomes, and suggests that nuclear positioning in trichomes depends on dynamic MTs (Mathur et al., 1999). While nuclear ploidy is vital for trichome development, the correlative relationship between trichome branching and nuclear placement might also be biologically relevant. Further study of the nucleus’ role in cellular branching may shed more light on trichome development.

### ROOT HAIR TIP GROWTH

Fundamentally, the tip-growing root hair has a similar organization between plant species. The root hair tip is depleted of cytoskeletal elements (Lloyd et al., 1987; Miller et al., 1999). Behind this is the “smooth” or “vesicle-rich” zone, which contains fine F-actin and traveling Golgi vesicles (Miller et al., 1999). Further from the tip is the cytoplasmic dense region (CDR), which is populated by F-actin, endoplasmic microtubules (EMTs), and the nucleus. Beyond the CDR, the root hair is largely vacuolated. During tip growth, the nucleus maintains a constant distance from the tip of 77 ± 15 μm in *Arabidopsis*, 30 ± 5 μm in *Medicago truncatula* (*Medicago*), and 45 ± 10 μm in *Vicia hirsuta* (Vetch; Lloyd et al., 1987; Sieberer and Emons, 2000; Ketelaar et al., 2002). At the end of tip growth, the nucleus disengages from the root hair tip and moves to a random location within the root hair cell (Sieberer and Emons, 2000).

Optical trapping of *Arabidopsis* root hair nuclei indicates that root hair growth depends on maintenance of nucleus-tip distance through constant nuclear movement. Stalling the nucleus leads to CDR disappearance and a growth termination-like cytoarchitectural shift (Ketelaar et al., 2002). Upon releasing the optical trap, the nucleus shifts toward the base of the root hair. This result is strikingly similar to the cytoarchitecture shift and growth termination observed when subapical fine F-actin is selectively depolymerized (Ketelaar et al., 2002). Additionally, backward movement of the nucleus following fine F-actin depolymerization was dependent on both F-actin and protein synthesis. This led to the hypothesis that nuclear movement in tip-growing *Arabidopsis* root hairs is solely actin-mediated, supported by recent findings that the actin motor Myosin XI-i is at least partially responsible for nuclear positioning and shape in mature root hairs (Ketelaar et al., 2002; Tamura et al., 2013). MTs appear to play no role in nuclear movement in *Arabidopsis* root hairs, though their depolymerization does lead to morphological changes (Bibikova et al., 1999; van Bruaene et al., 2004).

In contrast, the effects of selective depolymerization of *Medicago* EMTs strongly resemble the effects of depolymerizing *Arabidopsis* subapical fine F-actin, including a slower growth rate, a growth termination-like cytoarchitectural shift, and an increase in tip-nucleus distance (Ketelaar et al., 2002; Sieberer et al., 2002). In *Medicago*, after the nucleus moves basipetally in response to EMT depolymerization, it returns to following the root hair tip, but at an increased distance (Sieberer et al., 2002). In Vetch, this basipetal movement depends on actin filaments, as in *Arabidopsis* (Lloyd et al., 1987; Sieberer et al., 2002). Depolymerization of actin, whether general or specific to subapical fine F-actin, halted tip growth but did not affect nuclear positioning (Lloyd et al., 1987; Miller et al., 1999).

Thus, while the actin-mediated mechanism for both basipetal and acropetal nuclear movement is conserved between *Arabidopsis* and legumes, the mechanism for tethering the nucleus to the tip has diverged. Curiously, this specialization does not appear to originate in any cytoarchitectural difference, though the nucleus follows the growing root hair tip more closely in legumes. One explanation might lie in the nucleus’ involvement in nodulation initiation in legumes. As discussed in the next section, MTs are also involved in connecting the nucleus to the infection thread (IT) tip.

## NUCLEAR MOVEMENT IN BIOTIC INTERACTIONS

### SYMBIOSIS

Nodulation is the intracellular accommodation by legumes of bacteria known as rhizobia (reviewed in Gage, 2004). Rhizobia produce Nod factors, which signal the plant to begin the process of bacterial invasion: the root hair tip swells, and the nucleus moves from a random position within the root hair to a location near the tip (Sieberer and Emons, 2000). A new outgrowth subsequently forms, which the nucleus enters as the root hair curls around and traps extracellular rhizobia (Sieberer and Emons, 2000). At this point, the nucleus disengages from the root hair tip and moves to a site proximal to the bacteria, and the IT begins to form. The IT is a tubular plasma membrane and cell wall invagination in which rhizobia grow and divide, and is connected to the nucleus by dense MTs (Fåhraeus, 1957; Timmers et al., 1999). IT development follows the path of the moving nucleus directly, regardless of its direction, supporting the idea that nuclear movement is necessary for IT guidance (Fåhraeus, 1957). As the root hair nucleus and IT move toward the body of the root, cortical cells in their path undergo cytoplasmic reorganization, where the nucleus moves from the cell periphery to the center, and cytoplasmic strands aggregate around it (Bakhuizen, 1988). This results in a bridge of cytoplasmic and cytoskeletal elements called the pre-infection thread (PIT; Bakhuizen, 1988). PITs allow the plant to dictate where the IT grows, and further emphasizes the role of the nucleus in nodulation (**Figure 1**; van Brussel et al., 1992).

Arbuscular mycorrhizal symbiosis (AMS) is more widespread than nodulation, occurring in 70–90% of land plant species (Smith and Read, 2008). While mainly symbionts, arbuscular mycorrhizal fungi (AMF) can also form parasitic interactions with *Arabidopsis* roots if a host plant is nearby (Veiga et al., 2013).

When communication is initiated, the plant releases strigolactones, and the fungus responds by producing Myc factors, the fungal equivalent of Nod factors (reviewed in Harrison, 2012). The fungal hypha grows toward and contacts the root, forming an appressorium, where the hypha spreads across an epidermal cell (Giovannetti et al., 1994). That cell’s nucleus moves to the contact site, at which point the pre-penetration apparatus (PPA), a donut-shaped structure of MTs and endoplasmic reticulum (ER), forms around it (Genre et al., 2005). The nucleus subsequently moves to the cortical side of the cell, leaving a tunnel of PPA material behind it. This tunnel guides the growth of the hypha as it penetrates the cell via membrane invagination (Genre et al., 2005). Similar to the PIT in nodulation, a PPA forms in cortical cells in the hypha’s path, creating a continuous tunnel (**Figure 1**; Genre et al., 2008). While the nucleus’ position predicts the site of PPA formation, no causative relationship has been established between nuclear movement and AMS. Ideally, future studies will reveal a nuclear movement factor that can be removed without affecting overall cytoskeletal organization, which would allow investigation of the nucleus’ role in symbiosis without perturbation of overall cellular architecture.

### PLANT–PATHOGEN INTERACTIONS

The body of research on the nucleus’ role in pathogenesis is relatively broad, but most research has been on oomycetes. When an oomycete comes in contact with a resistant plant cell, that cell’s nucleus moves to the pathogen contact site, followed by local wall thickening and cytoplasmic aggregation (**Figure 1**; Guest, 1986; Freytag et al., 1994; Daniel and Guest, 2005). If these steps avert infection, the nucleus moves away from the contact site. If the oomycete continues to form a haustorium and enter the incompatible cell, the nucleus and a thick aggregation of cytoplasm become associated with it, followed by hypersensitive cell death (Guest, 1986; Freytag et al., 1994). In a compatible interaction, however, the nucleus does not move to the contact site (**Figure 1**). It may later become associated with the haustorium, but this association has no apparent impact on infection outcome (Guest, 1986; Freytag et al., 1994; Daniel and Guest, 2005). These data suggest that the nucleus may play a vital role in plants’ response to oomycete pathogens.

Recently, Caillaud et al. (2012) observed plant nuclei near developing haustoria in susceptible *Arabidopsis* mesophyll cells, seemingly contradicting the above model. However, their study differs from those cited above in that they observed mesophyll cells 4 days after infection, whereas the older studies generally observed epidermal cells within 24 h. It is therefore possible that mesophyll cell nuclei respond differently to oomycete pathogens than epidermal cell nuclei, or that the nucleus becomes associated with the haustorium after a longer period of time.

Unlike in oomycete response, the plant nucleus moves to the site of contact during both compatible and incompatible interactions with fungi (Heath et al., 1997). If hyphal penetration is successful, the nucleus moves away from the contact site, whereas if penetration is unsuccessful, the nucleus remains at the contact site. If the hypha penetrates a resistant cell, the plant cell initiates hypersensitive cell death. However, if a hypha penetrates a susceptible cell, the nucleus associates with the hypha, though to what end is still unclear (Heath et al., 1997). This pattern of the nucleus moving toward, then away from, the contact site mimics the actions of the nucleus during intracellular accommodation of symbiotic fungi (Genre et al., 2005). Similarly, a nodulation mimic also exists. The nematode *Meliodogyne incognita* produces a factor that causes root hair deformation similar to that caused by exogenous application of Nod factors, including cytoplasmic reorganization and nuclear movement reminiscent of nodulation initiation (Weerasinghe et al., 2005). The significance of this phenomenon for the life cycle of the nematode is not well-understood, but the parallels are intriguing, and provide further evidence for a long-held theory that symbiosis and parasitism share an evolutionary and mechanistic history (Gollotte et al., 1993).

## NUCLEAR MOVEMENT IN ABIOTIC SIGNALING

### RESPONSE TO BLUE LIGHT

Light-dependent nuclear movement was first observed in the fern *Adiantum capillus-veneris*, where prothallus nuclei moved from anticlinal walls toward a white light source, and return to the anticlinal walls following dark adaptation (Blatt et al., 1981; Kagawa and Wada, 1993; reviewed in Takagi et al., 2011; Higa et al., 2013). The light-dependent movement from anticlinal to periclinal walls was caused by blue and red light (Kagawa and Wada, 1995; Tsuboi et al., 2007). In *Arabidopsis* leaves, mesophyll cell nuclei move from periclinal to anticlinal walls and epidermal cell nuclei move to convex regions in response to blue light (**Figure 1**; Iwabuchi et al., 2007). This response is mediated by the blue light receptor Phototropin 2 (PHOT2) in an actin-dependent manner (Iwabuchi et al., 2007; Iwabuchi and Takagi, 2010). Additionally, nuclear repositioning in response to cold stress was observed in *Adiantum capillus-veneris* and the liverwort *Marchantia polymorpha L*. (Kodama et al., 2008; Ogasawara et al., 2013).

Interestingly, Myosin XI-i mutants defective in root hair nuclear movement also are defective in dark-induced nuclear movement, but not blue light-induced nuclear movement (Tamura et al., 2013). Myosin XI-i interacts with WIT1 and WIT2, *Arabidopsis* outer nuclear envelope proteins that associate with the KASH proteins WIP1, WIP2, and WIP3 (Zhao et al., 2008; Zhou et al., 2012; Tamura et al., 2013). This suggests that the mechanism for nuclear movement during dark adaptation may differ from that for light adaptation. As the function of light-responsive nuclear movement is not well-understood, further study into the requirement for actin reorganization independent of PHOT2 activation may be necessary to understand how nuclei move in leaf cells.

### RESPONSE TO MECHANICAL STIMULI

Nuclear movement in response to mechanical stimuli was first described as actin-dependent movement of the nucleus to a wound site (Katsuta and Shibaoka, 1988; Nagai, 1993; Williamson, 1993; reviewed in Takagi et al., 2011). Deformation of the cell wall and plasma membrane was initially proposed to induce nuclear movement to the stimulation site (Kennard and Cleary, 1997). However, later studies in several species showed that stimulation with a 2–10 μm needle could induce nuclear movement to the stimulus site and cellular rearrangement of actin, MTs, and ER (Gus-Mayer et al., 1998; Qu and Sun, 2007; Hardham et al., 2008; Genre et al., 2009). Mechanical stimulation of the *Medicago* AMS defective mutant *dmi3* leads to cell death, rather than nuclear movement, suggesting a shared mechanism between AMS and mechanical stimulus response (Genre et al., 2009).

In tobacco leaf epidermal cells, nuclei move dynamically from one mechanical stimulation site to the next, without any lag time or decrease in velocity (Qu and Sun, 2007). The nucleus is first connected to the stimulus site by a cytoplasmic strand, followed by movement toward the stimulation site and cytoskeletal reorganization (Qu and Sun, 2008a, b). While the pathway sensing mechanical stress has yet to be characterized in plants, it has been suggested that the nucleus may act as an organizing center for responses to mechanical stimuli (Qu and Sun, 2008b). If this is the case, nuclear movement to sites of stimulation by pathogens, symbionts, and abiotic factors may have the same function- by moving the nucleus to the stimulus site, the plant can respond more efficiently to further perturbation.

## OUTLOOK

The examples discussed above outline that nuclear positioning in plants can be observed in conjunction with critical phases of development, signaling, and biotic interactions. However, often neither the functional relevance, nor the molecular mechanisms are well established. The role of the actin or MT cytoskeleton has been well documented in several cases. In animals, the critical role of nuclear positioning during development is achieved through nuclear envelope-anchored SUN-KASH protein complexes (Starr and Fridolfsson, 2010). The recent discovery of the first plant KASH family–WIP–represents a potential starting point for uncovering the mechanism of nuclear positioning in plants (Zhou et al., 2012). Although WIPs are not responsible for nuclear positioning in trichomes or root hairs (Zhou et al., 2012), they might be involved in nuclear positioning in other tissues. This discovery also implies that more KASH families could remain unidentified in plants.

## Conflict of Interest Statement

The authors declare that the research was conducted in the absence of any commercial or financial relationships that could be construed as a potential conflict of interest.
